# Supplementary material for: Utilizing the Off-Target Effects of T1R3 Antagonist Lactisole to Enhance Nitric Oxide Production in Basal Airway Epithelial Cells
Source: Nutrients. 2023 Jan 19;15(3):517. doi: 10.3390/nu15030517 (PMC9919013; doi:10.3390/nu15030517)
Supplement: Supplementary file 1 [file nutrients-15-00517-s001.zip › nutrients-2135155-supplementary.pdf]

Supplementary Figures

Cell Lines Used	Source	Catalogue Number
Beas-2B	ATCC	CRL-9609
Primary human bronchial epithelial cells	Lonza	CC-2450
Primary nasal epithelial cells	This Study	N/A
**Chemicals Used**	**Source**	**Catalogue Number**
(-)-a-Thujone	Millipore Sigma	89231
8-pCPT-2'-O-Me-cAMP-AM	Tocris	4853
ATP	Millipore Sigma	A9187
Bovine Serum Albumin	Millipore Sigma	A2153
Caffeine	Millipore Sigma	C0750
CellEvent Caspase 3/7 Reagent	ThermoFisher Scientific	C10423
Crystal Violet	Millipore Sigma	C0775
DAF-FM diacetate	ThermoFisher Scientific	D23844
Denatonium Benzoate	Millipore Sigma	D5765
Diphenhydramine	Cayman Chemical	11158
ER-Tracker Green	Cell Signaling Technologies	8787S
ESI-09	Cayman Chemical	19130
Flufenamic Acid	Millipore Sigma	F9005
Fluo-8 AM	Abcam	ab142773
Forskolin	Millipore Sigma	F3917
Fura-2 AM	ThermoFisher Scientific	F1221
H89 Dihydrochloride	Tocris	2910/1
Histamine	Millipore Sigma	H7125
Isoproterenol	Millipore Sigma	I6504
Lactisole	Cayman Chemical	18657
Lipofectamine 3000	ThermoFisher Scientific	L3000075
MEM Amino Acids	ThermoFisher Scientific	11130051
Phenylthiocarbamide (PTC)	Millipore Sigma	P7629
Quinine	Millipore Sigma	Q0132
SNAP (S-Nitroso-N-acetylpenicillamine)	Cayman Chemical	82250
Thapsigargin	Cayman Chemical	10522
XTT Reagent	ThermoFisher Scientific	X6493
YM-254890	Cayman Chemical	29735
**Recombinant DNA - Function**	**Source**	**Catalogue Number**
4mtD3cpv - mitochondrial Ca2+ biosensor	Addgene	36324
AKAR4 - intracellular PKA activity	Addgene	61619
AKAR4-nls - nuclear PKA activity	Addgene	138217
Epac-pH187 - intracellular cAMP; EPAC cAMP binding domain	Addgene	170338
Flamindo2 - intracellular cAMP	Addgene	73938
nes-R-GECO - non-nuclear Ca2+	This Study	N/A
nls-Flamindo2 - nuclear cAMP	Addgene	73939
nls-R-GECO - nuclear Ca2+	Addgene	32462
**Taqman Probes for qPCR**	**Source**	**Catalogue Number**
ATP2A1	ThermoFisher Scientific	Hs01092295_m1
ATP2A2	ThermoFisher Scientific	Hs00544877_m1
ATP2A3	ThermoFisher Scientific	Hs01024563_m1
ITPR1	ThermoFisher Scientific	Hs00181881_m1
ITPR2	ThermoFisher Scientific	Hs00181916_m1
ITPR3	ThermoFisher Scientific	Hs01573539_m1
RYR1	ThermoFisher Scientific	Hs00166991_m1
RYR2	ThermoFisher Scientific	Hs00181461_m1
RYR3	ThermoFisher Scientific	Hs00168821_m1
TAS1R1	ThermoFisher Scientific	Hs01547926_g1
TAS1R2	ThermoFisher Scientific	Hs00541095_m1
TAS1R3	ThermoFisher Scientific	Hs00877446_g1
UBC	ThermoFisher Scientific	Hs01871556_s1

Table S1. A list of reagents used in this study.

Bitter Compound	Bitter Taste Receptors Activated	Citation
Denatonium Benzoate	T2R4, T2R8, T2R10, T2R13, T2R39, T2R43, T2R46, T2R47	[1,2]
Diphenhydramine	T2R14, T2R40	[1,2]
Flufenamic Acid	T2R14	[1,2]
PTC	T2R38	[1,2]
Quinine Sulfate	T2R4, T2R7, T2R10, T2R14, T2R39, T2R40, T2R43, T2R44, T2R46	[1,2]
Thujone	T2R10, T2R14	[1,2]

Table S2. Bitter compounds and their associated T2Rs.

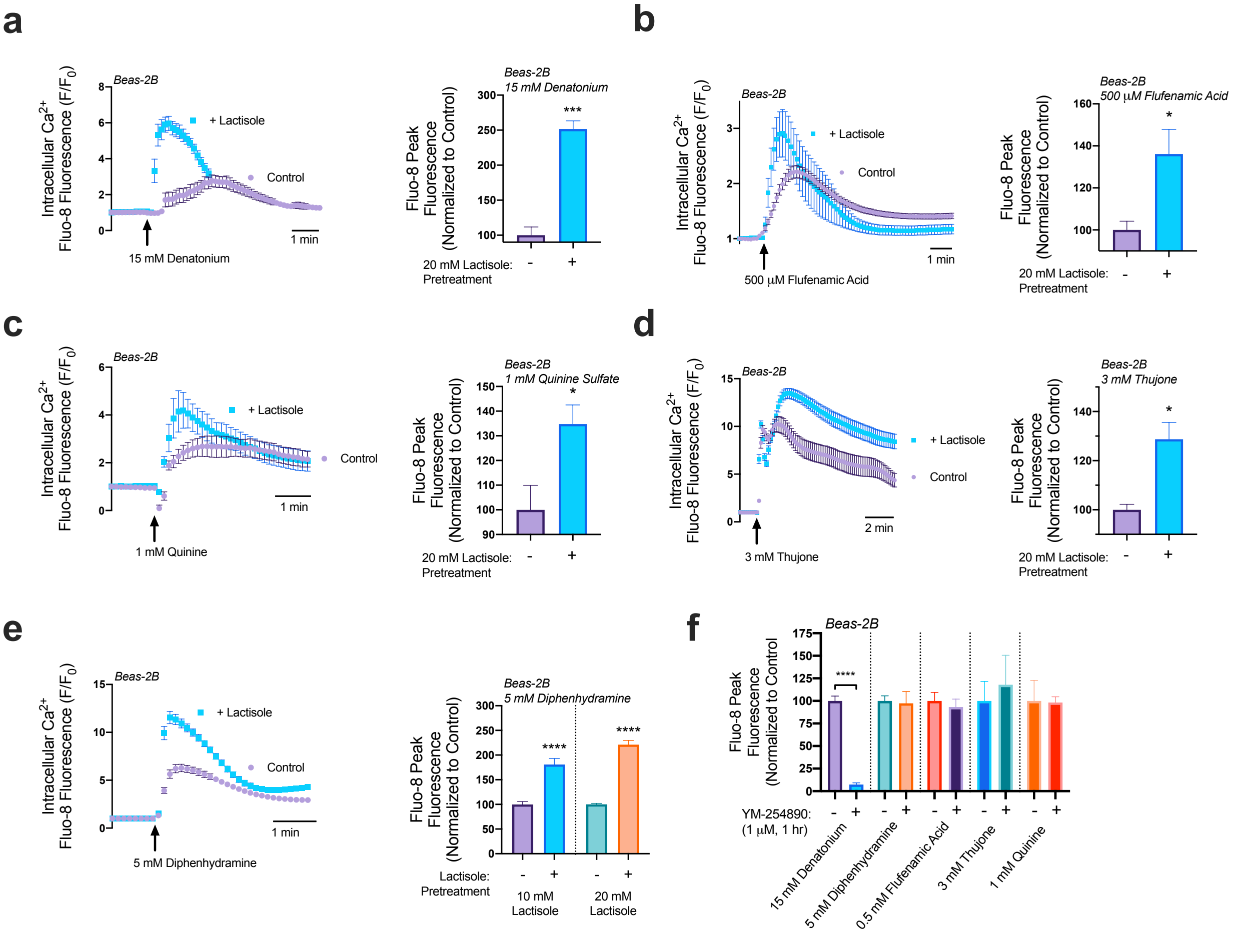

Figure S1. Lactisole enhanced Ca^{2+} elevations from many bitter compounds. A one-hour pretreatment with 20 mM lactisole increased Ca^{2+} elevations from (**a**) 15 mM denatonium treatment 250%, (**b**) 500 uM flufenamic acid treatment by 35%, (**c**) 1 mM quinine sulfate treatment by 35%, (**d**) 3 mM thujone treatment by 30%, and (**e**) 5 mM diphenhydramine treatment by 220%. A one-hour pretreatment with 10 mM lactisole increased diphenhydramine Ca^{2+} elevations by 180%. (**f**) Pretreatment of Beas-2B's with 1 µM of YM-254890 inhibited 15 mM denatonium-signaled Ca^{2+} elevations by 93% but had no effect on any other bitter compound. Bar graphs containing two comparisons were analyzed via t-test $*P < 0.05$, $***P < 0.001$, $****P < 0.0001$, 'n.s.' represents no significance.

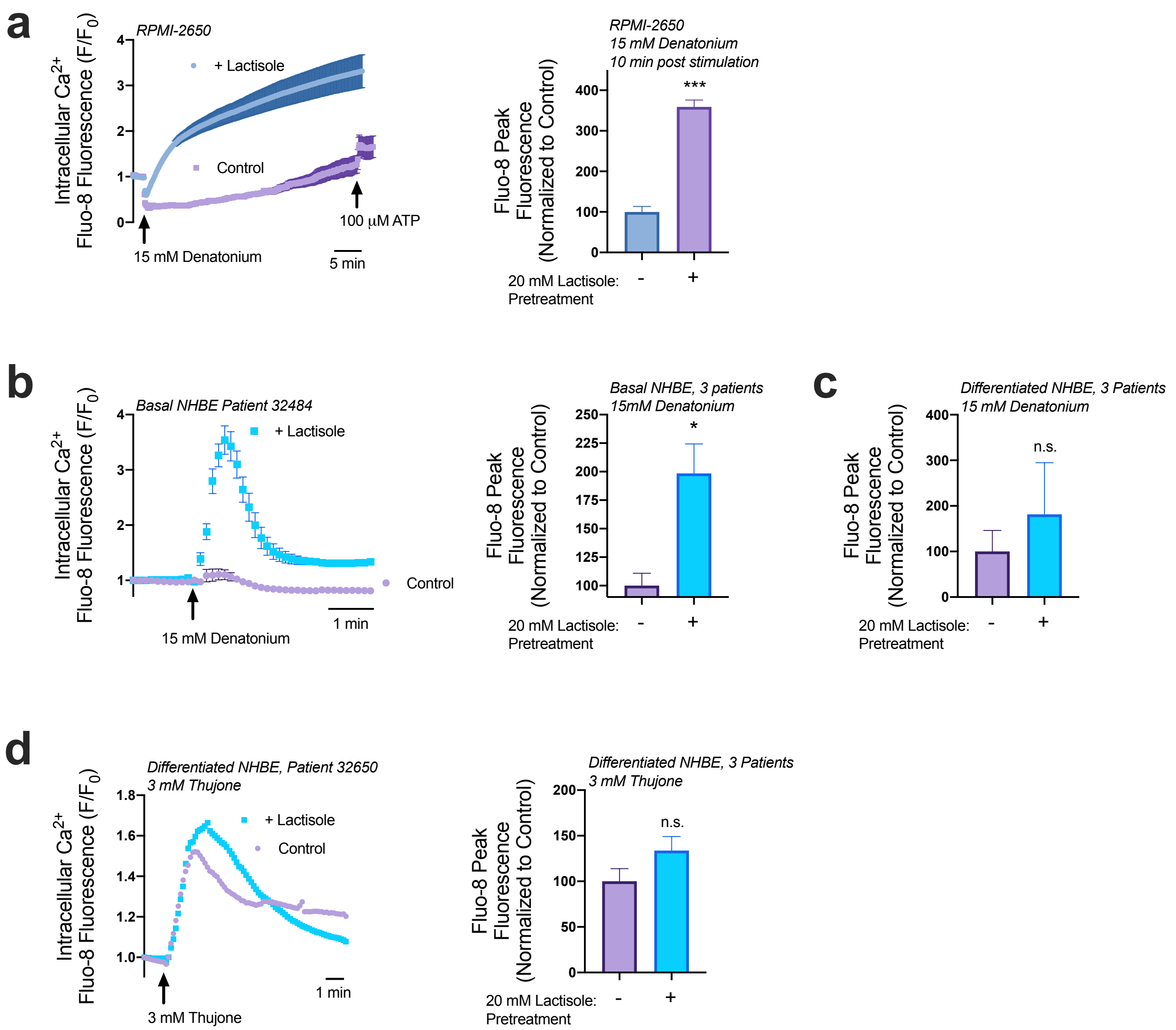

Figure S2. Lactisole enhanced Ca^{2+} signaling in basal epithelial cell cultures. (**a**) Squamous cell carcinoma line RPMI-2650 only displayed intracellular Ca^{2+} release via 15 mM denatonium treatment when pretreated with 20 mM lactisole for 1 hour. (**b**) Pretreatment of primary basal NHBE's with 20 mM lactisole for 1 hour enhanced denatonium-induced Ca^{2+} release by 200%. (**c**) Fully differentiated primary NHBE's did not respond to 15 mM denatonium treatment via intracellular Ca^{2+} signaling pathways, regardless of lactisole pretreatment (20 mM, 1 hr). (**d**) Fully differentiated primary NHBE's signal via intracellular Ca^{2+} in response to 3 mM thujone treatment but did not display an enhancement of Ca^{2+} signaling with lactisole pretreatment (20 mM, 1 hr). Bar graphs containing two comparisons were analyzed via t-test $*P < 0.05$, $***P < 0.001$, 'n.s.' represents no significance.

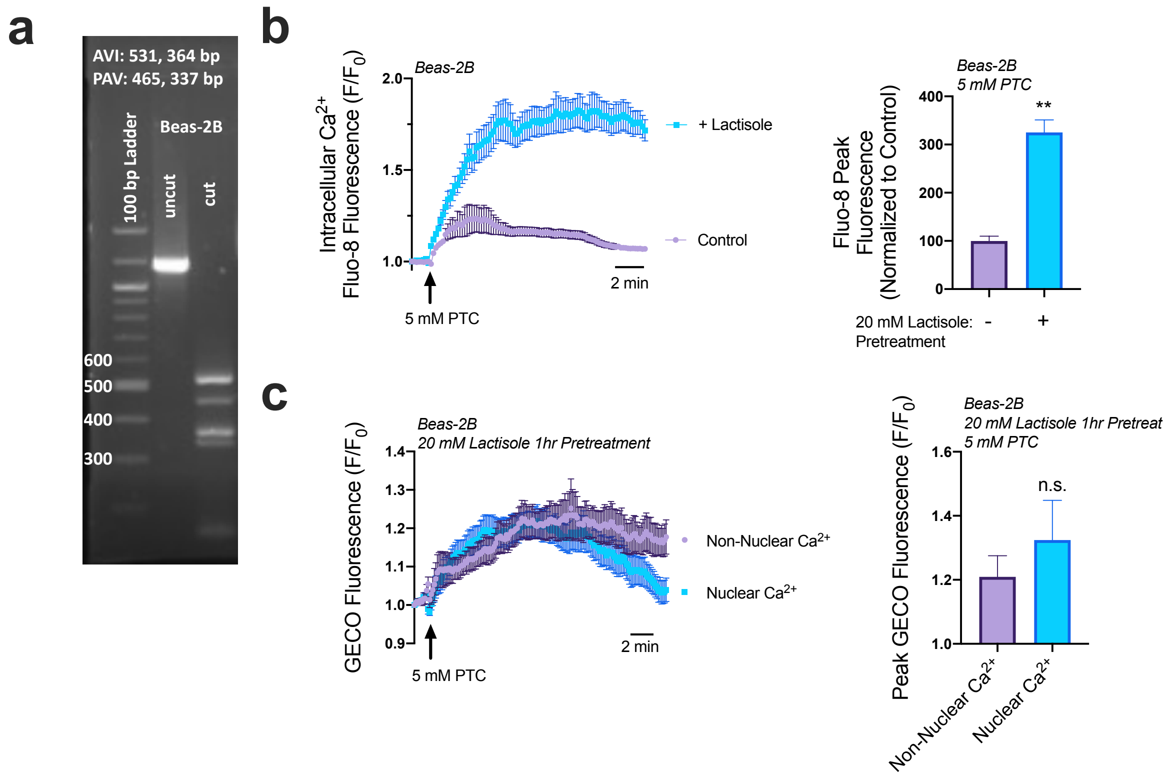

Figure S3. Lactisole increases PTC-signaled mitochondrial membrane depolarization. (**a**) Beas-2B genomic T2R38 cDNA was digested with FNu4HI for 1hr (37°C). Presence of bands at 364 and 531 bp's displayed presence of T2R38 AVI allele while bands at 337 and 465 bp's revealed presence of T2R38 PAV allele. (**b**) 5 mM PTC induced mild elevations in intracellular Ca^{2+} in Beas-2B cells. PTC-signaled Ca^{2+} elevations increased by 300% with 20 mM lactisole pretreatment (1 hr). (**c**) Beas-2B cells pretreated with 20 mM lactisole for 1 hour displayed both nuclear and non-nuclear Ca^{2+} elevations in response to 5 mM PTC. Bar graphs containing two comparisons were analyzed via t-test $**P < 0.01$, 'n.s.' represents no significance.

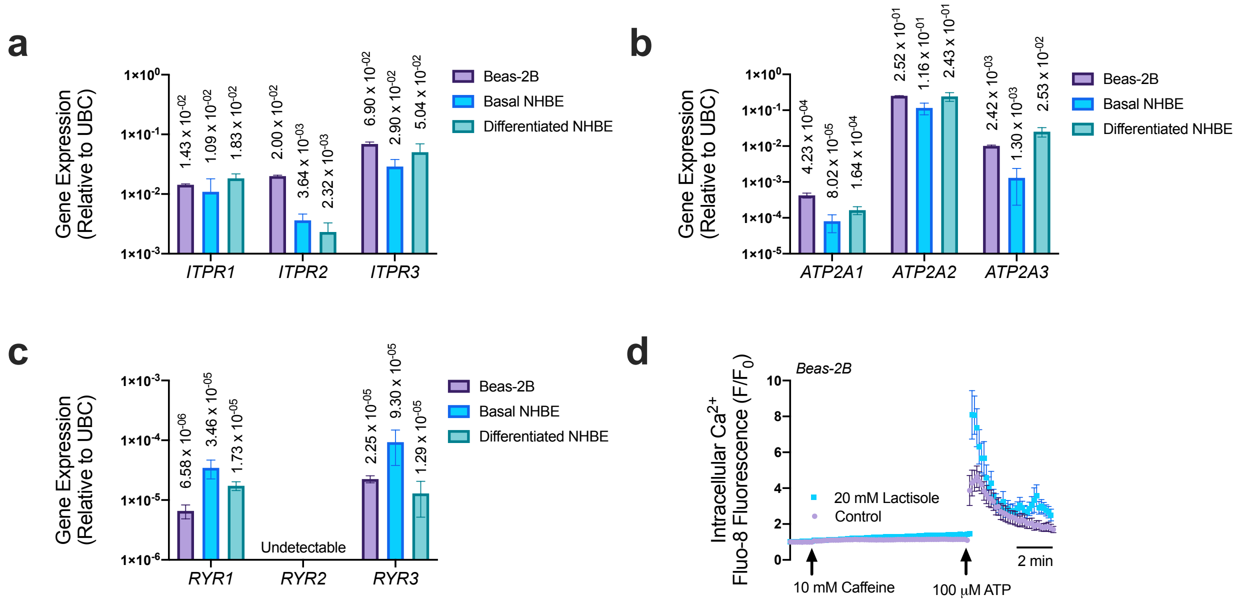

Figure S4. *IP3*, *RYR*, and *SERCA* expression in airway epithelial cells. Beas-2B's, basal normal human bronchial epithelial cells, and differentiated normal human bronchial epithelial cells (NHBE's; >21 days at air) contained comparable levels of (**a**) *ITPR1, ITPR2, ITPR3,* coding for three different inositol triphosphate receptors (**b**) *ATP2A1, ATP2A2, ATP2A3* coding for three different sarcoendoplasmic reticulum calcium ATPases, and (**c**) *RYR1, 2, 3* coding for three different ryanodine receptors. (**c**) All cells tested lacked expression of ryanodine receptor 2 (*RYR2*). (**d**) Airway epithelial cell ryanodine receptor transcript expression was notably low. To test for ryanodine receptor activity, Beas-2B cells were loaded with Ca^{2+} chelating dye Fluo-8 and treated with 10 mM caffeine with or without a pretreatment of 20 mM lactisole to amplify Ca^{2+} signaling pathways. No Ca^{2+} elevations were observed with caffeine treatment regardless of lactisole treatment.

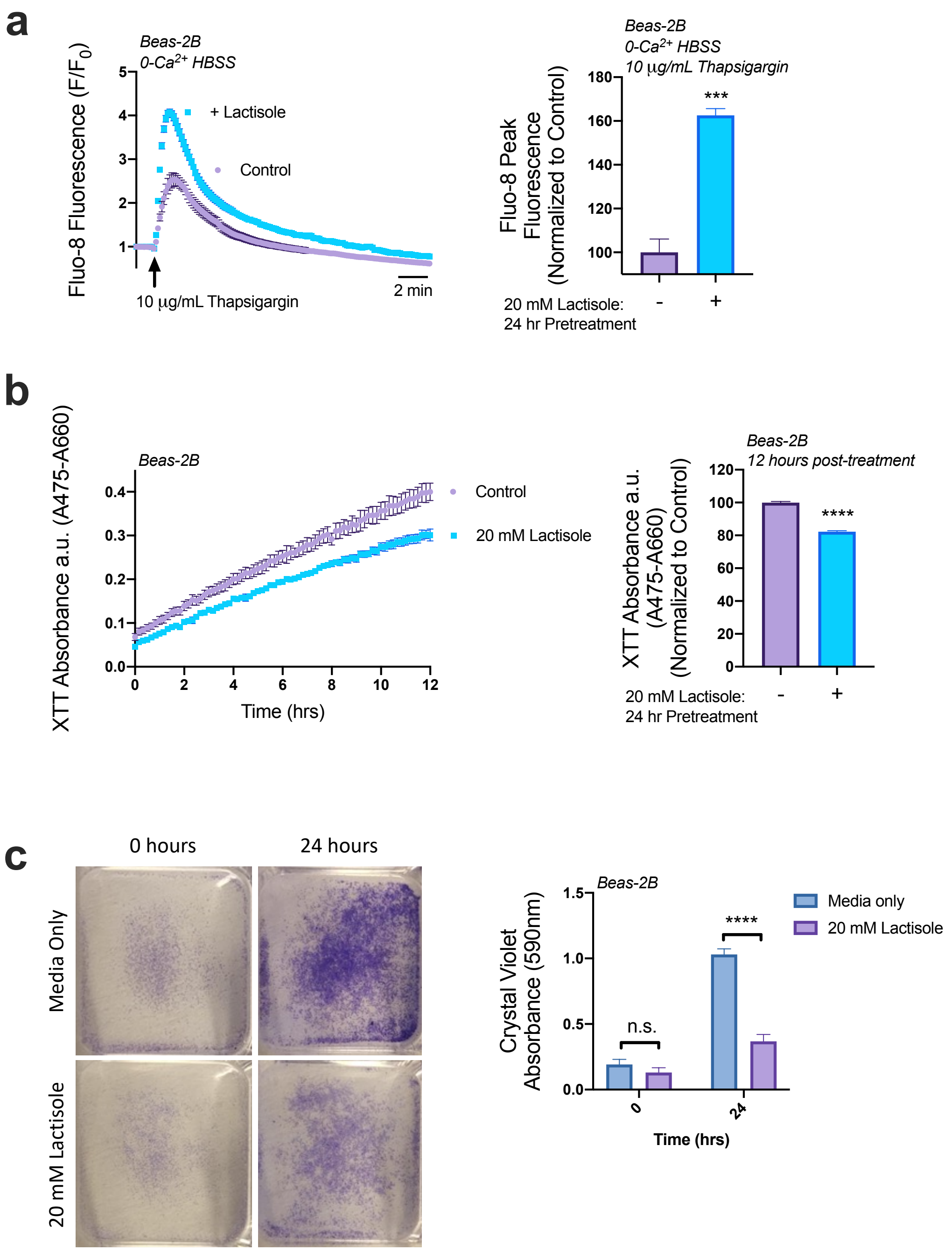

Figure S5. The long-term effects of lactisole treatment. (**a**) 24-hour treatment of Beas-2B's with 20 mM lactisole increased ER Ca^{2+} content by 60%. (**b**) Pretreatment of Beas-2B's with 20 mM lactisole for 24 hours reduced cell viability as visualized through XTT absorbance by 20%. (**c**) An equal amount of Beas-2B's were seeded 16 hours prior to treatment with 20 mM lactisole for 24 hours. Lactisole treatment notably slowed the proliferation of Beas-2B cells as visualized by protein/DNA stain crystal violet. Bar graphs containing only two comparisons were analyzed via t-test; the bar graph with >2 comparisons were analyzed using ANOVA with Sidak's multiple comparison posttest ***$P < 0.001$, ****$P < 0.0001$, 'n.s.' represents no significance.

References

1. Dagan-Wiener, A.; Di Pizio, A.; Nissim, I.; Bahia, M.S.; Dubovski, N.; Margulis, E.; Niv, M.Y. BitterDB: Taste Ligands and Receptors Database in 2019. *Nucleic Acids Res* **2019**, *47*, D1179–D1185.
2. Meyerhof, W.; Batram, C.; Kuhn, C.; Brockhoff, A.; Chudoba, E.; Bufe, B.; Appendino, G.; Behrens, M. The Molecular Receptive Ranges of Human TAS2R Bitter Taste Receptors. *Chem Senses* **2010**, *35*, 157–170.
